# In-vitro antiproliferative efficacy of *Abrus precatorius* seed extracts on cervical carcinoma

**DOI:** 10.1038/s41598-022-13976-7

**Published:** 2022-06-17

**Authors:** Amritpal Kaur, Yash Sharma, Anoop Kumar, Madhumita P. Ghosh, Kumud Bala

**Affiliations:** 1 Therapeutics and Molecular Diagnostic Lab, Center for Medical Biotechnology, Amity Institute of Biotechnology, Amity University, Noida, Uttar Pradesh India; 2 National Institute of Biologicals, Noida, India

**Keywords:** Biochemistry, Biotechnology, Cancer, Drug discovery, Biomarkers

## Abstract

*Abrus precatorius* is a tropical medicinal plant with multiple medicinal benefits whose seeds have not yet been studied against cervical cancer. Herein, we have assessed the antioxidant and antiproliferative properties of seed extracts (ethyl acetate and 70% ethanol) prepared from Soxhlet and Maceration extraction methods against Hep2C and HeLa Cells. We observed that the APE (Sox) extract had a significantly higher total flavonoid content, APA (Mac) extract had a high total phenolic content, and APA (Sox) extract had a high total tannin content. Further, HPLC analysis of extracts revealed the presence of tannic acid and rutin. Moreover, APA (Sox) exhibited the highest free radical scavenging activity. APE (Mac) had the best antiproliferative activity against Hep2C cells, while APA (Sox) had the best antiproliferative activity against HeLa cells. In Hep2C cells, APE (Mac) extract revealed the highest SOD, catalase activity, GSH content, and the lowest MDA content, whereas APA (Mac) extract demonstrated the highest GST activity. In HeLa cells, APA (Sox) extract showed the highest SOD, GST activity, GSH content, and the least MDA content, whereas APA (Mac) extract showed the highest catalase activity. Lastly, docking results suggested maximum binding affinity of tannic acid with HER2 and GCR receptors. This study provides evidence that *A. precatorius* seed extracts possess promising bioactive compounds with probable anticancer and antioxidant properties against cervical cancer for restricting tumor growth.

## Introduction

Cancer is one of the major causes of mortality in both men and women worldwide and its research has garnered attention from the scientific fraternity all across the globe. Considering the global burden of gynecological cancers, cervical cancer (CaCx) among them ranks as the fourth most common cancer with nearly 604,000 new reported cases and 342,000 deaths in 2020^1^. Despite effective screening measures and treatment modalities, cervical cancer continues to hold the banner of the leading cause of cancer related mortality among women^2^. Therefore, the development of novel therapeutic drugs with increased efficiency is warranted. Interestingly, receptors like Glucocorticoids (GCR), Human epidermal growth factor receptor 2 (HER2), Vascular endothelial growth factor (VEGF), estrogen and progesterone receptors are reported to be associated with progression of cervical cancer^3^. Moreover, high levels of estrogen and progesterone facilitate hormonal imbalance in women and therefore, the above-mentioned receptors have been established as promising therapeutic candidates as corroborated by in-silico studies^4–7^. Out of the 1881 new chemical entities that were approved as drugs between 1981 and 2019, purely synthetic compounds accounted for only 24.6%, while the majority of the new drugs were derived from medicinal plants^8^. Herbal plants have made important contributions to the development of anticancer drugs. Numerous natural substances are recognized to be antioxidants, cancer preventive agents or even antitumor agents such as paclitaxel^9^.

*Abrus precatorius* L. (Fabaceae) is a plant that spans tropical and subtropical parts of the world. Lowly elevated, dry regions are conducive to its growth. Known for its medicinal value, its leaves, roots and seeds are often exploited for anti-helminthic, anti-diarrheal, neuroprotective, anti-depression, anti-fertility, anti-cataract, anti-arthritic, anti-allergic and anti-emetic purposes. *Abrus* derived lectins have been widely used in treating various cancers^10^. Evidence advocates that the seeds of this medicinal herb are effective in treating diabetes and chronic nephritis. Moreover, *A. precatorius* leaves possess a sweetness quotient equivalent to sucrose and are therefore used to sweeten foods in West tropical Africa^11^.

The main aim of the study was to prepare *A. precatorius* seed extracts using Soxhlet and Maceration methods to identify various phytochemical compounds and evaluate their abilities to function as antioxidants and as antiproliferative agents in human cervix carcinoma cells.

## Results

### Molecular identification

**Figure 1:**
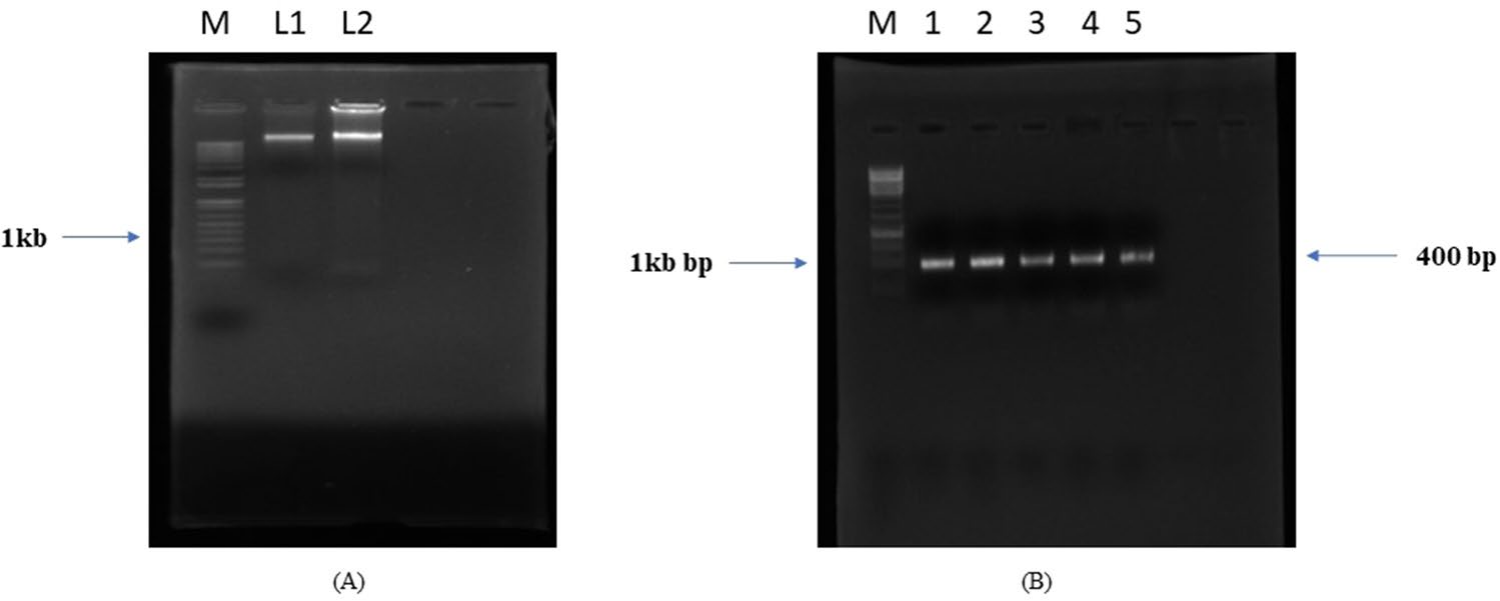
(**A**) represents M; 1 kb Marker, L1 and L2 (gDNA replicates), and (**B**) represents M; 1 kb Marker, 1,2,3,4 and 5; Amplicon replicates (~ 400 bp).

Genomic DNA was isolated from seeds and was run on 0.8% agarose gel. A gel documentation system was used for viewing the gel. Genomic DNA was then used as a template for amplification of a conserved gene for species identification. PCR amplification was done using *rbcLa*-forward and *rbcLa*-reverse primers. A product of around 400 base pairs was obtained (Fig. 1). The PCR product was then run on 1.2% agarose gel followed by elution from the gel. The sample was utilized for sequencing using *rbcLa*-forward and *rbcLa*-reverse primers. The NCBI BLAST_N program was used to compare the nucleotide sequence available in GenBank. Sequence analysis confirmed the sample showed 98.8% identity having 81% query cover with previous reported sequences of *A. precatorius* (NC_047402.1).

### Quantification of phytochemicals constituents

**Figure 2:**
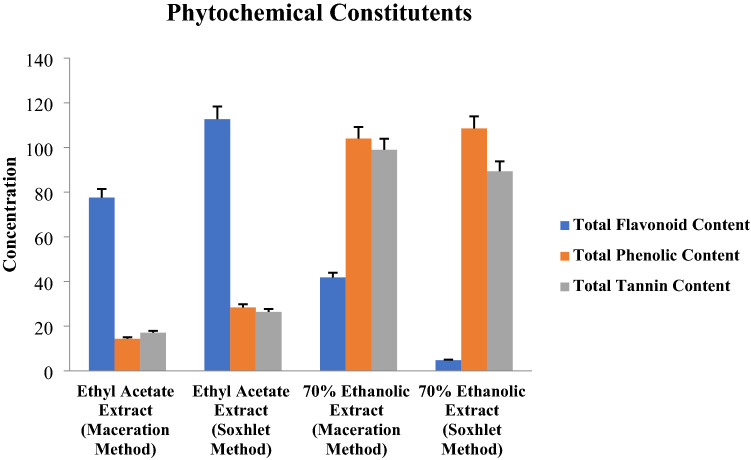
Comparative quantitative analysis of phytochemical constituents found in seed extracts prepared with 70% ethanol and ethyl acetate employing soxhlet and maceration extraction methods.

Total flavonoid content in *A. precatorius* seed extracts was obtained using a standard calibration curve (y = 0.010x, r^2^ = 0.984) of quercetin (20–100 µg/mL) and expressed in mg quercetin equivalents/g of extract as shown in Fig. 2. APE (Sox) extract demonstrated high flavonoid content i.e., 112.16 ± 0.9 mg quercetin equivalents/g of extract, while APE (Mac) extract showed 76.86 ± 1.51 mg quercetin equivalents/g of extract. Moreover, APA (Mac) extract exhibited 41.88 ± 0.77 mg quercetin equivalents/g of extract while APA (Sox) extract only showed 4.79 ± 0.1 mg quercetin equivalents/g of extract (Fig. 2) (Graph 1, Supplementary).

Similarly, the phenolic content of *A. precatorius* seed extracts was determined using a standard calibration curve (y = 0.015x, r^2^ = 0.998) of quercetin (20–100 µg/mL) and expressed in mg quercetin equivalents/g of extract as shown in Fig. 2. Phenolic content of APA (Mac) and APA (Sox) seed extracts showed a similar trend, i.e., 108.88 ± 0.4 mg quercetin equivalents/g of extract and 103.96 ± 0.17 mg quercetin equivalents/g of extract, respectively. Whereas, APE (Sox) and APE (Mac) seed extracts showed less amount of quercetin equivalents at 28.53 ± 0.41 mg/g of extract and 14.71 ± 0.39 mg/g of extract, respectively (Fig. 2) (Graph 2, Supplementary).

Total tannins content of *A. precatorius* seed extracts was estimated using a standard calibration curve (y = 0.0143x, r^2^ = 0.998) of tannic acid (20–100 µg/mL) and expressed in mg tannic acid equivalents/g of extract (Fig. 2). The results showed that APA (Sox) seed extract had 98.98 ± 1 mg tannic acid equivalents/g of extract, while APA (Mac) seed extract had 89.41 ± 0.67 mg tannic acid equivalents/g of extract. APE (Sox) and APE (Mac) seed extracts, on the other hand, had less tannic acid equivalents, with 26.15 ± 0.18 mg/g extract and 17.10 ± 0.13 mg/g extract, respectively (Fig. 2) (Graph 3, Supplementary).

### Antioxidant activity

**Figure 3:**
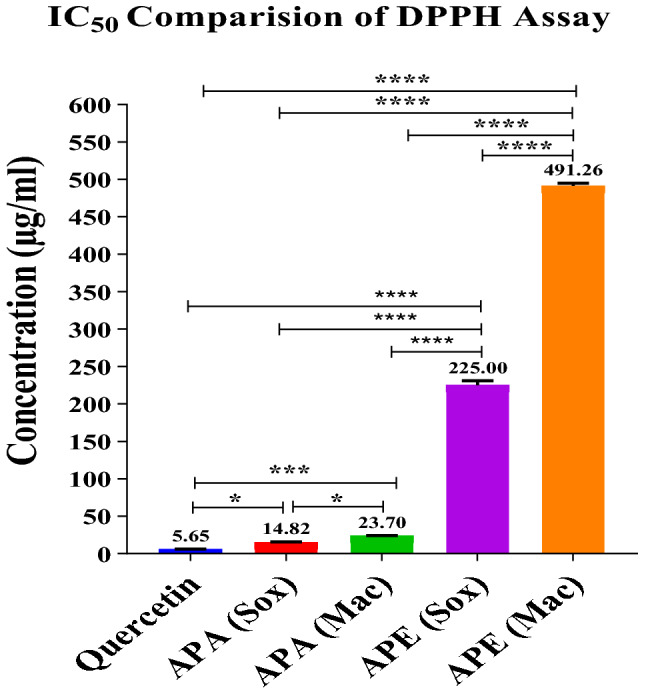
DPPH radical-scavenging activity: The comparison of the IC_50_ values of *A. precatorius* seed extracts; APA (Sox) (1–21 µg/mL), APA (Mac) (1–29 µg/mL), APE (Sox) (150–170 µg/mL), APE (Mac) (150–650 µg/mL), and quercetin (1–21 µg/mL) at various concentrations. The significance between various groups was tested using one-way ANOVA where (*) *p* = 0.0308, (***) *p* = 0.0003, (****) *p* < 0.0001.

The antioxidant potential of *A. precatorius* seed extracts were analyzed by DPPH free radical scavenging assay. Quercetin (standard) and the different seed extracts showed variable antioxidant properties. The lower IC_50_ value indicates higher radical scavenging activity. The order of radical scavenging activity was APA (Sox) > APA (Mac) > APE (Sox) > APE (Mac). APA (Sox) with an IC_50_ value of 14.82 ± 0.85 µg/mL showed the highest radical scavenging activity among other extracts as shown in Table 1 and Fig. 3 (Graph 4, Supplementary). The standard (quercetin) exhibited an IC_50_ value of 5.65 ± 0.29 µg/mL.

**Table 1:** IC_50_ values obtained in DPPH assay and FRAP values.

	IC_50_ (µg/mL) *	(mM Fe (II)/g dry weight of extract) *
Sample	DPPH	FRAP values
APA (Mac)	23.70 ± 0.43	105,026.4 ± 57.50
APA (Sox)	14.82 ± 0.85	95,601 ± 413.96
APE (Mac)	491.26 ± 3.54	6230.39 ± 24.31
APE (Sox)	225.00 ± 5.92	5866.97 ± 39.41

Values are means of three replicate samples (n = 3). Data are presented as the mean ± SD.

Antioxidant capacity was also quantified by FRAP assay. FRAP values were obtained using a standard calibration curve (y = 0.001x, r^2^ = 0.919) of FeSO_4_ (100–1000 µM) and evaluated based on the capacity to reduce ferric (III) iron to ferrous (II) iron. The results were expressed in mM Fe (II) equivalents/g dry weight of seed extract. APA (Mac) had the maximum FRAP value (105,026.4 ± 57.50 mM Fe (II)/g dry weight of seed extract) among other extracts as shown in Table 1. (Graph 5, Supplementary).

### HPLC analysis

**Figure 4:**
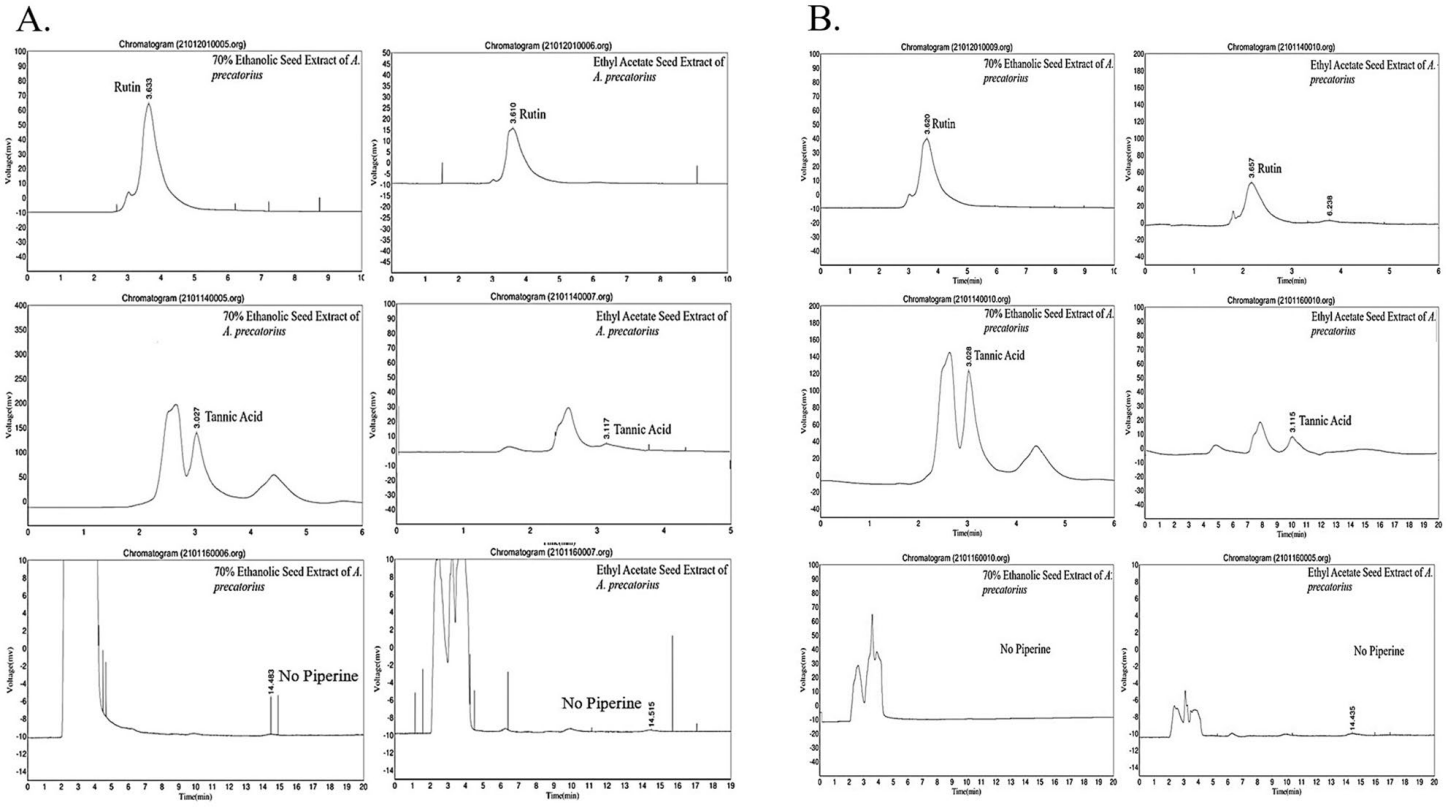
HPLC Chromatograms: (**A**); 70% ethanolic and ethyl acetate seed extracts of *A. precatorius* prepared from soxhlet extraction method, (**B**); 70% ethanolic and ethyl acetate seed extracts of *A. precatorius* prepared from maceration extraction method.

HPLC was performed to measure the polyphenolic flavonoids (rutin), tannins (tannic acid), and alkaloids (piperine) content of *A. precatorius* seed extracts prepared using different extraction methods. The retention times for the standards (rutin, tannic acid, and piperine) were RT 3.81, RT 3.09, and RT 14.84, respectively. Furthermore, rutin and tannic acid were identified in APA (Sox), APA (Mac), APE (Sox), and APE (Mac) seed extracts and their retention times were similar to the standards (rutin, tannic acid, and piperine). However, piperine was not identified in any of the extracts, as shown in (Fig. 4). The identified compounds and their corresponding quantities are listed in Table 2. From the above results, APA (Mac) extract had the highest concentrations of tannic acid (369.54 ± 1.26 mg/g of dry weight of extract), whereas APA (Sox) had the highest rutin concentration (333.44 ± 1.51 mg/g of dry weight of extract). Further, in context to the ethyl acetate extract, a similar trend was observed where APE (Mac) exhibited high concentrations of rutin (70.62 ± 0.69 mg/g of dry weight of extract) and tannic acid (175.03 ± 0.66 mg/g of dry weight of extract). Contrary to the above observations, piperine was not exhibited by any of the extracts as proven by the standard curve calibration results (Graph 6–8, Supplementary).

**Table 2:** Polyphenolic flavonoids and alkaloid content in *A. precatorius* seed extracts.

Extracts	Identified compounds (mg/g of dry weigh of extract) *		
	Rutin	Tannic acid	Piperine
APA (Mac)	235.55 ± 5.31	369.54 ± 1.26	ND
APA (Sox)	333.44 ± 1.51	345.02 ± 0.15	ND
APE (Mac)	70.62 ± 0.69	175.03 ± 0.66	ND
APE (Sox)	63.73 ± 0.77	77.24 ± 0.46	ND

*Values are means of three replicate samples (n = 3). Data are presented as the mean ± SD. ND; Not Detected.

### Cytotoxic activity of seed extracts against cervical cancer cells

**Figure 5:**
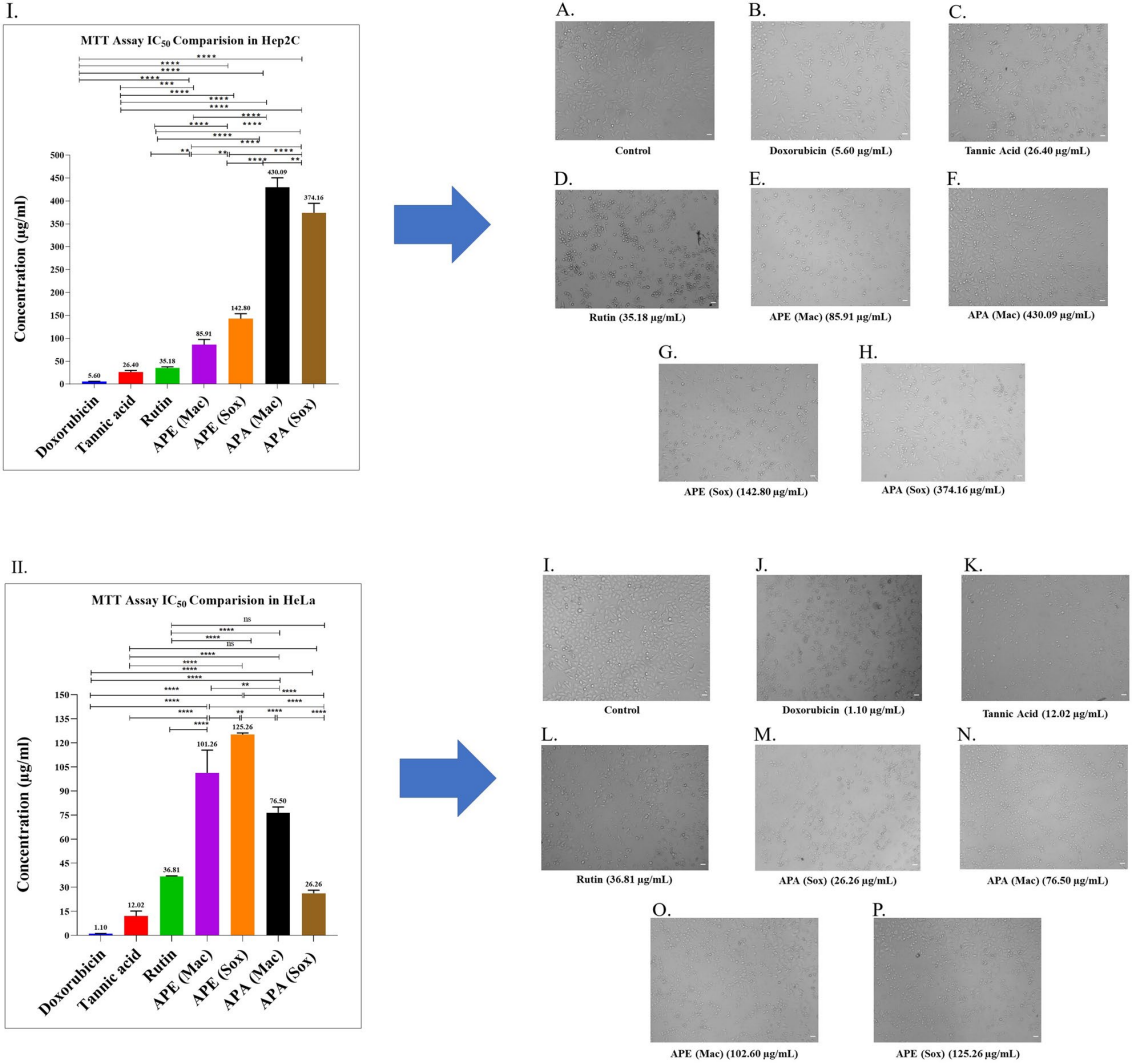
Antiproliferative activity on cervical cancer cells (Hep2C and HeLa): **(I–II)**; The comparison of average IC_50_ between *A. precatorius* seed extracts and standards (doxorubicin, rutin and tannic acid) over 48 h (Y-axis: concentration in (µg/mL)). The significance between various groups was tested using one-way ANOVA where: ns; non-significant, (*) *p* = 0.0308, (** *p* = 0.0014), (***) *p* = 0.0003, (****) *p* < 0.0001. **(A–H)**; Morphological changes showing inhibition of Hep2C using IC_50_ specific values of *A. precatorius* seed extracts and standards after 48 h**. (I–P)**; Morphological changes showing inhibition of HeLa using IC_50_ specific values of *A. precatorius* seed extracts and standards after 48 h. The cells were evaluated under an Olympus inverted microscope using 10X objective lens (magnification 100X). Scale bar: 100 μm.

*A. precatorius* seed extracts were evaluated for their antiproliferative potential using MTT assay. Doxorubicin, tannic acid, rutin, and *A. precatorius* seed extracts all had varying antiproliferative efficacy, with a dose-dependent reduction in cell viability of cervical cancer cells (Hep2C and HeLa) (Supplementary (Graph 9–10). IC_50_ values were also determined, lower IC_50_ values indicating higher the antiproliferative activity. In Hep2C cells, the order of cytotoxicity was APE (Mac) > APE (Sox) > APA (Sox) > APA (Mac). APE (Mac) with an IC_50_ value of 85.91 ± 6.7 µg/mL, showed particularly high antiproliferative activity among other extracts as shown in Table 3 and Fig. 5. Additionally, Doxorubicin, rutin and tannic acid demonstrated IC_50_ values of 5.60 ± 0.29 µg/mL, 35.18 ± 1.46 µg/mL and 26.40 ± 1.85 µg/mL, respectively. Similarly, in HeLa cells, the order of cytotoxicity was found to be APA (Sox) > APA (Mac) > APE (Mac) > APE (Sox). APA (Sox) with an IC_50_ value of 26.26 ± 1.09 µg/mL, showed particularly high antiproliferative activity among other extracts as shown in Table 3 and Fig. 5. Further, Doxorubicin, rutin and tannic acid demonstrated IC_50_ values of 1.10 ± 0.07 µg/mL, 36.81 ± 0.17 µg/mL and 12.02 ± 1.82 µg/mL, respectively. Morphological changes in Hep2C and HeLa were observed after 48 h of treatment with the IC_50_ values of *A. precatorius* seed extracts. In comparison to untreated cells, most of the Hep2C and HeLa cells changed from spindle to star-shaped, with some becoming damaged and shrunk (Fig. 5). Meanwhile, their growth was inhibited in a concentration-dependent manner.

**Table 3:** In-vitro the antiproliferative activity (IC_50_) of extracts of *A. precatorius* seeds after 48 h of treatment.

IC_50_ (µg/ml) *		
Sample	Hep2C	HeLa
Doxorubicin	5.60 ± 0.29	1.10 ± 0.07
Tannic acid	26.40 ± 1.85	12.02 ± 1.82
Rutin	35.18 ± 1.46	36.81 ± 0.17
APE (Mac)	85.91 ± 6.7	101.26 ± 8.21
APE (Sox)	142.80 ± 6.24	125.26 ± 0.51
APA (Mac)	430.09 ± 11.81	76.50 ± 2.01
APA (Sox)	374.16 ± 11.85	26.26 ± 1.09

*Values are means of three replicate samples (n = 3). Data are presented as the mean ± SD.

### Antioxidant enzymes activity assay on cells

The antioxidant enzyme activity (SOD, CAT, and GST) as well as non-enzyme content (GSH and MDA) of *A. precatorius* seed extracts on Hep2C and HeLa cells were measured using IC_50_ specific values to estimate the intracellular reduction of reactive oxygen species (ROS).

### Superoxide dismutase activity

**Figure 6:**
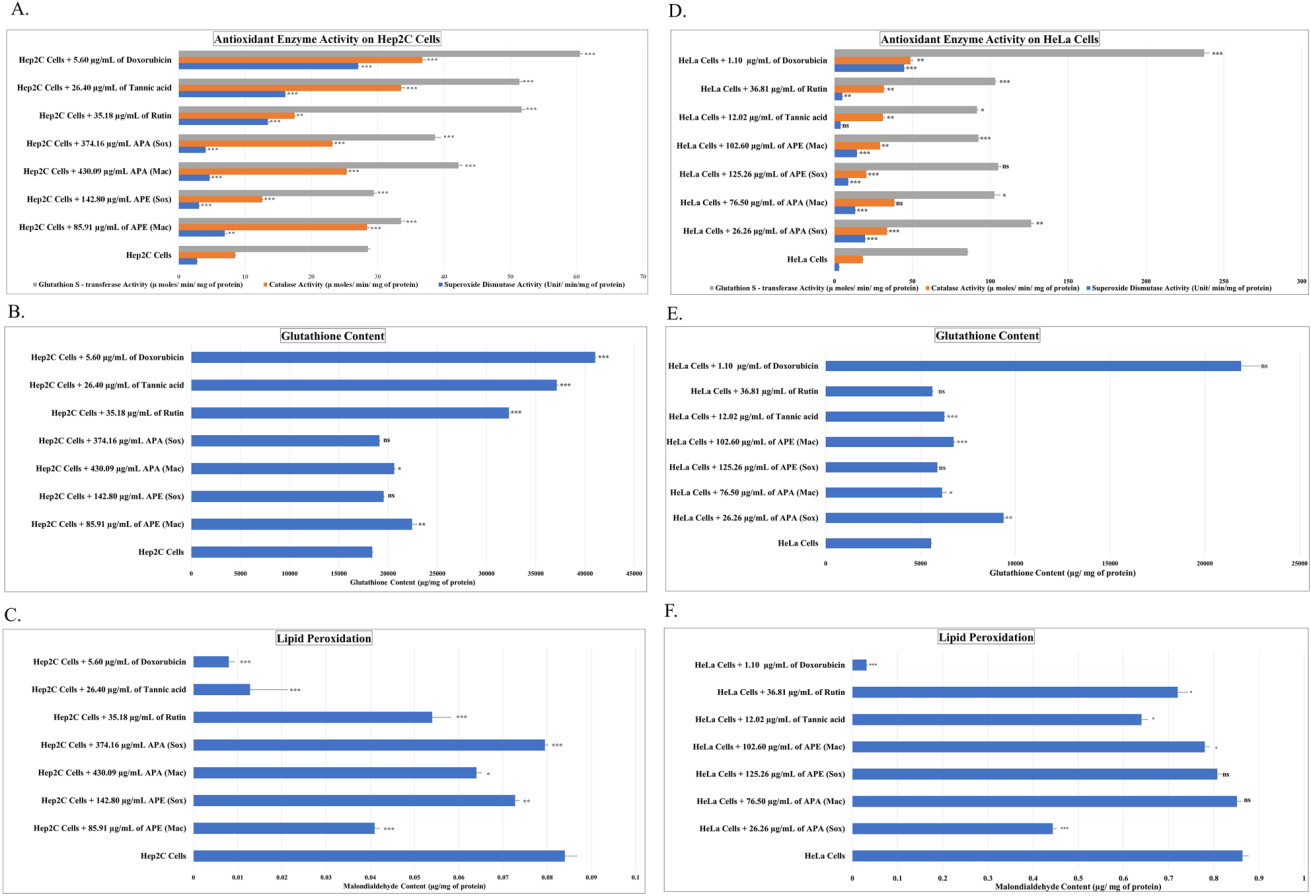
Antioxidant enzyme activity of *A. precatorius* seed extracts on Hep2C and HeLa cells illustrating **(A)**; SOD, CAT & GST activity in Hep2C Cells, **(B–C)**; non-enzyme content (GSH and MDA) in Hep2C Cells, **(D)**; SOD, CAT & GST activity in HeLa Cells and **(E–F)**; non-enzyme content (GSH and MDA) in HeLa Cells. The data is representative of three consecutive experiments. * *p* < 0.05, ** *p* < 0.01, *** *p* < 0.001, ns = non-significant.

As shown in Fig. 6A, the highest SOD enzymatic activity was observed in APE (Mac) extract (6.92 ± 0.24 U/min/mg of protein) as compared to the control cells (2.74 ± 0.02 U/min/mg of protein). SOD activity of APA (Mac) and APA (Sox) was found to be (4.63 ± 0.03 U/min/mg of protein) and 4.07 ± 0.03 U/min/mg of protein, respectively. APE (Sox) has the lowest enzyme activity (3.04 ± 0.02 U/min/mg of protein). Doxorubicin exhibited a significantly higher SOD activity (27.02 ± 0.02 U/min/mg of protein) compared to other compounds such as rutin and tannic acid, which exhibited 13.41 ± 0.05 U/min/mg of protein and 16.05 ± 0.03 U/min/mg of protein, respectively, as given in Fig. 6A. Similarly, in HeLa Cells, APA (Sox) extract had the highest SOD activity (9.68 ± 0.12 U/min/mg protein) compared to APE (Sox) (8.79 ± 0.09 U/min/mg protein). Furthermore, as compared to tannic acid (3.91 ± 0.76 U/min/mg of protein) and rutin (5.07 ± 0.14 U/min/mg of protein), doxorubicin showed the highest SOD activity (44.67 ± 0.91 U/min/mg of protein) (Fig. 6D).

### Catalase activity

Similar to above, the catalase enzyme activity was also observed to be the highest in the APE (Mac) extract (28.37 ± 0.01 µmoles/min/mg of protein) compared to the control i.e., 8.49 ± 0.06 µmoles/min/mg of protein and other extracts i.e., 23.15 ± 0.06 µmoles/min/mg of protein in APA (Sox) and 12.57 ± 0.03 µmoles/min/mg of protein in APE (Sox). Hep2C cells treated with doxorubicin had high catalase activity (36.72 ± 0.47 µmoles/min/mg of protein), followed by tannic acid (33.53 ± 0.01 µmoles/min/mg of protein) and rutin (17.49 ± 0.02 µmoles/min/mg of protein), as shown in Fig. 6A.

Furthermore, in HeLa cells treated with APA (Mac), the maximal catalase activity was reported to be (38.44 ± 1.11 µmoles/min/mg of protein), whereas rutin and tannic acid exhibited similar enzymatic activity. Following catalase activity, APA (Sox) and APE (Sox) displayed 23.69 ± 0.52 µmoles/min/mg of protein and 20.47 ± 1.28 µmoles/min/mg of protein, respectively. Interestingly, upon doxorubicin treatment, the catalase enzyme activity was revealed to be the highest i.e., 48.76 ± 2.85 µmoles/min/mg of protein (Fig. 6D).

### Glutathione S-transferase activity

The GST activity in the Hep2C cells upon treating with the APA (Mac) extract was observed to be the highest (42.16 ± 0.55 µmoles/min/mg of protein) compared to the control (28.5 ± 0.05 µmoles/min/mg of protein), followed by APA (Mac) and APE (Sox) i.e., 29.37 ± 0.04 µmoles/min/mg of protein and 38.56 ± 0.06 µmoles/min/mg of protein, respectively. Doxorubicin treatment resulted in maximum GST activity i.e., 60.46 ± 0.02 µmoles/min/mg of protein in comparison to rutin and tannic acid (Fig. 6A). In HeLa cells, treatment with different extracts and standards revealed that APA (Sox) had the highest GST activity (126.35 ± 2.58 µmoles/min/mg of protein), followed by APE (Sox) (105.10 ± 3.29 µmoles/min/mg of protein. Doxorubicin treatment resulted in the highest GST activity (237.35 ± 5.98 µmoles/min/mg of protein) in comparison to rutin and tannic acid as shown in Fig. 6D.

### Glutathione content

The non-enzyme content such as glutathione (GSH) was also quantified in the cells in a similar manner as above. Upon quantitative analysis, we found that among all the extracts, APE (Mac) treated Hep2C cells showed maximum glutathione content of 22,435.65 ± 5.76 µg/mg of protein compared to the control (18,363.86 ± 57.90 µg/mg of protein) (Fig. 6B). GSH content in APE (Sox) and APA (Sox) was found to be 19,539.64 ± 5.80 µg/mg of protein and 19,116.55 ± 2.18 µg/mg of protein, respectively. Similarly, HeLa cells treated with APA (Sox) had the maximum GSH content (9386.98 ± 78.17 µg/mg of protein) with respect to the control cells (5560.33 ± 37.50 µg/mg of protein). Furthermore, doxorubicin treatment resulted in the maximum GSH content (21,908.57 ± 1903.93 µg/mg of protein) compared to rutin (5629 ± 81.50 µg/mg of protein) and tannic acid (6284.38 ± 58.01 µg/mg of protein) as shown in Fig. 6E).

### Lipid peroxidation

Malondialdehyde content (MDA) was also estimated and Hep2C cells showed a marked decrease in the MDA content upon exposure to APE (Mac) (0.041 ± 0.89 µg/mg of protein) compared to the control cells i.e., 0.084 ± 0.12 µg/mg of protein (Fig. 6C). APE (Sox) and APA (Sox) had also shown very less MDA content i.e., 0.072 ± 0.08 µg/mg of protein and 0.079 ± 0.01 µg/mg of protein, respectively. On the other hand, in HeLa cells, the MDA content was observed to be minimal when exposed to APA (Sox) i.e., 0.44 ± 0.01 µg/mg of protein as compared to untreated (control), rutin and tannic acid, although cells treated with doxorubicin had much lower MDA content i.e., 0.031 ± 0.16 µg/mg of protein (Fig. 6F).

### Molecular docking analysis

**Figure 7:**
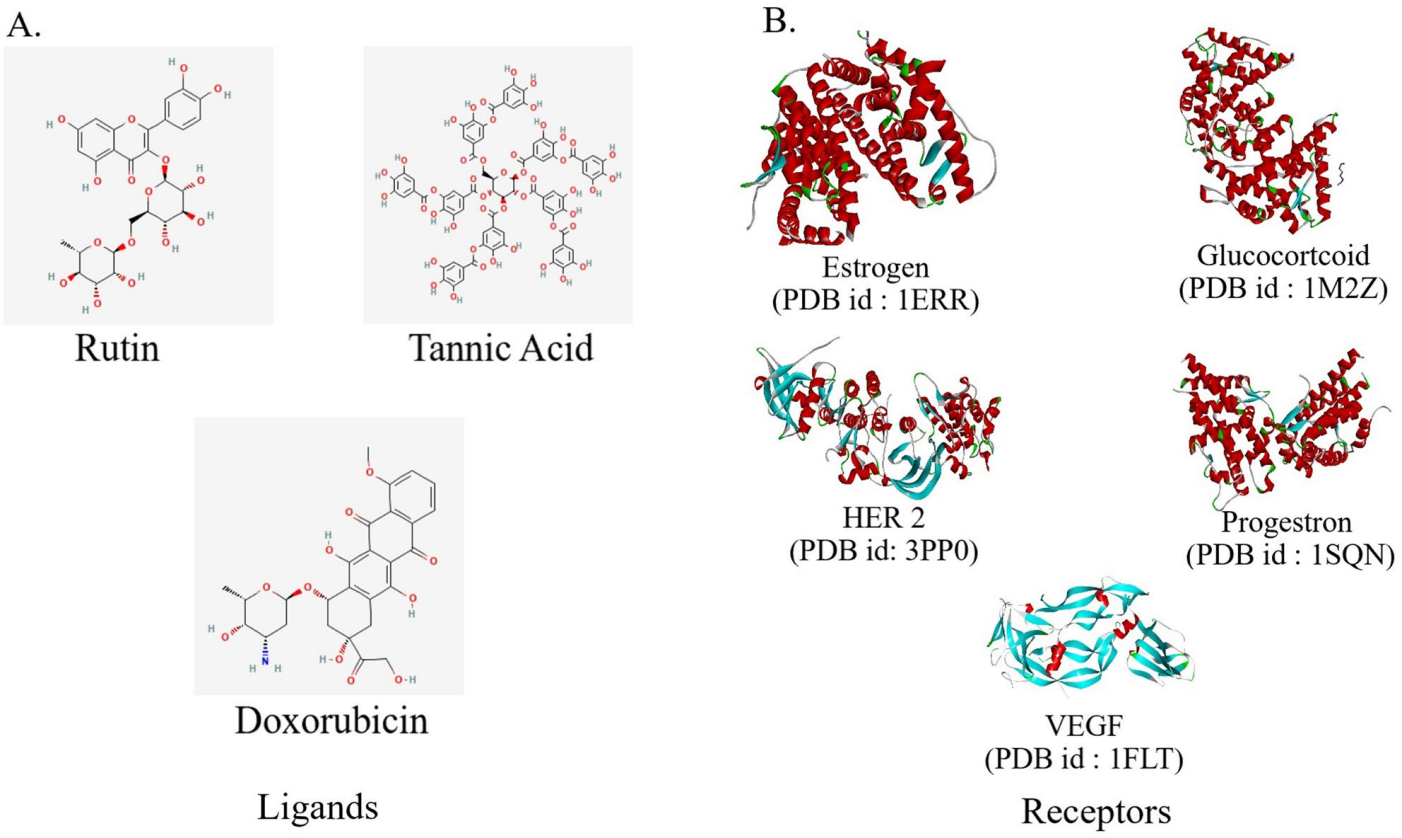
(**A**) Rutin, Tannic acid and Doxorubicin as ligands for molecular docking with (**B**) receptors of cervical cancer i.e., Estrogen (PDB ID: 1ERR), Glucocorticoid (PDB ID:1M2Z), HER 2 (PDB ID: 3PP0), Progesterone (PDB ID: 1SQN) and VEGF (PDB ID:1FLT).

**Figure 8:**
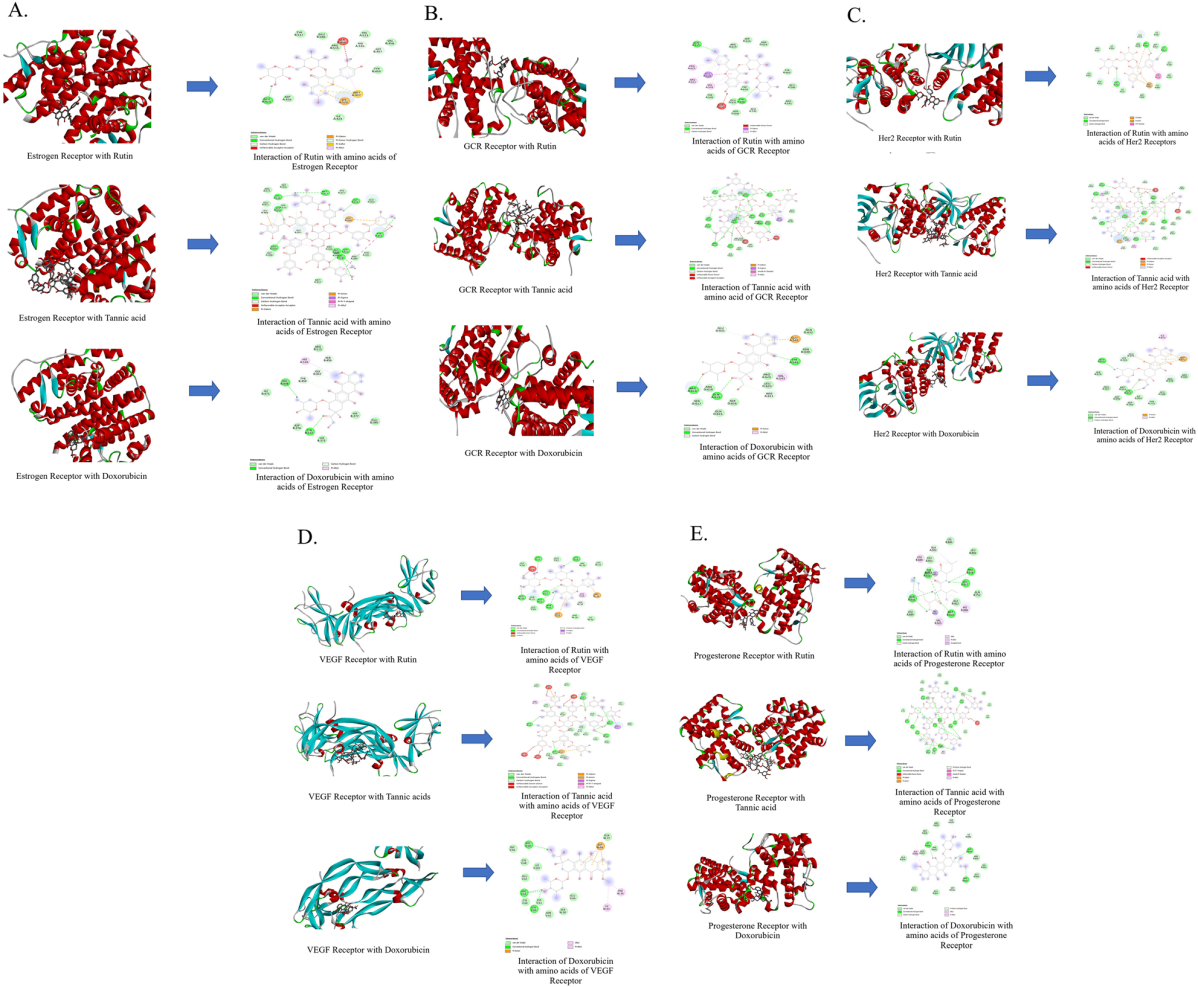
Molecular docking of Rutin, Tannic acid and their interaction with amino acids of (**A**) Estrogen receptor, (**B**) GCR receptor, (**C**) HER2 receptor, (**D**) VEGF receptor and (**E**) Progesterone receptor of cervical carcinoma.

In the docking analysis, the binding affinities of the identified compounds (Fig. 7A) were studied against the cervical cancer receptors including Estrogen, Progesterone, Glucocorticoid, VEGF and HER2 (Fig. 7B). The docking analysis revealed that tannic acid shared the maximum binding affinity with HER2 and GCR (− 9.1 kcal/mol and − 9.0 kcal/mol, respectively) as compared to doxorubicin, whereas rutin shared the strongest binding affinity with HER2 (− 8.9 kcal/mol) among other receptors such as estrogen, progesterone and VEGF (Table 4, Fig. 8). The docked complexes were further visualized for their molecular interactions using the Discovery Studio 2021 client as shown in Fig. 8.

**Table 4:** Estimated ΔG (Kcal/mol) of HPLC characterized compounds against cervical cancer targets.

Targets/Compounds	Tannic Acid	Rutin	Doxorubicin
Estrogen	− 6.7	− 7.1	− 6.9
GCR	− 9.0	− 8.4	− 7.8
HER2	− 9.1	− 8.9	− 7.8
Progesterone	− 8.6	− 7.6	− 8.3
VEGF	− 8.0	− 8.3	− 7.1

## Discussion

Cervical cancer remains a burden for women of low‑ and middle‑income countries (LMICs) such as India, South Africa, China and Brazil. In 2018, there were half a million new cases of cervical cancer and 311,365 deaths due to lack of adequate treatment^12^. Currently, the recommended therapeutic regimens include chemotherapy, radiation therapy, and surgery^13^. However, they present several limitations, including side effects or ineffectiveness. Therefore, it is important to search for novel therapeutic agents or drug candidates that are naturally synthesized which will specifically act on the cancer cells without affecting normal cells. Plant extracts and their bioactive compounds play a significant role in prevention of cancer and many more diseases^14^. Plants have proven to be an excellent reservoir of polyphenols, tannins, flavonoids, alkaloids, terpenes, etc*.*^15^. Recently, more attention has been placed on tannins with the utilization of some herbs such as *Phyllanthus emblica*, *Sanguisorba officinalis,* as well as red wine with considerable tannins^16^. Plant-derived chemotherapeutic agents such as cisplatin, carboplatin, paclitaxel, ifosfamide, curcumin, camptothecin, taxol, and combretastatin have been used widely against cervical carcinoma^17^. Keeping all the above points in mind, the present study was designed to evaluate ethyl acetate and 70% ethanol seed extracts of *A. precatorius* obtained by different extraction methods as a potential therapy against cervical carcinoma by evaluating their antioxidant activity and in-vitro anti-proliferative activity, as well as binding affinity of their polyphenolic flavonoids (rutin) and tannins (tannic acid) against receptors mediating signaling pathways in cervical carcinoma.

DNA barcoding is a technique that involves isolation of the genomic and/or organelle DNA and sequencing of a conserved region for species identification. It differs from molecular phylogeny in a way that the main goal is not to determine classification but to identify an unknown sample in terms of a known classification^18^. DNA barcode sequences are very short relative to the entire genome and they can be obtained reasonably quickly and cheaply^19^. Here, the plant sample was identified on the basis of DNA barcoding from the Consortium for the Barcode of Life (CBOL) database. Recently, this technique has been widely used for validating plant as well as animal identification. Various known markers for the identification of plants are *trnH*—*PsbA*, *ITS F*, *matK*, *rbcL*^20^. Other reports also suggested use of plant barcoding for the identification^19,20^ .

Here, we reported that the content of major flavonoids of *A. precatorius* was significantly higher in APE (Sox) seed extract (112.16 ± 0.9 mg quercetin equivalent/g of extract) as compared to the other extracts. Similarly, the total phenolic content in *A. precatorius* was higher in APA (Mac) seed extract (108.88 ± 0.4 mg quercetin equivalent/g of extract) in comparison to the other extracts. Moreover, the total tannin content of APA (Sox) amounting to 98.98 ± 1 mg tannic acid equivalents/g of extract was higher than that of other extracts. As corroborated previously, less content of flavonoids, phenolics and tannins of *A. precatorius* have been reported^23^. Another study reported that polarity of the solvents used for extraction plays an important role in the concentration of phenols and flavonoids^24–26^. Indeed, several factors, such as the type of solvent, the extraction process, the part of the plant, temperature, and so on, might influence the yield and extraction of phenolic compounds^27,28^.

It is well established that HPLC can be used to identify, separate and quantify phytochemicals^29^. In our investigation, HPLC analysis aimed at characterization of flavonoid compounds revealed an abundance of rutin in the APA (Sox) (333.44 ± 1.51 mg/g DW) extract whereas tannic acid was observed to be abundant in the APA (Mac) (369.54 ± 1.26 mg/g DW) extract. Literature cites that rutin was less abundant (24.13 ± 1.26 µg/g DW) in the leaves of *A. precatorius*^30^. However, to our knowledge, no studies pertaining to the quantification of tannic acid have been reported in *A. precatorius*, whereas prior studies on the preparation of 80% ethanolic extract of Quercus species has revealed very less tannin content i.e., 127.68 mg/g as compared to the present studies^31^.

Antioxidant capacity of the plant extracts mainly depends on both the composition of the extracts as well as the test system. It is influenced by a variety of factors and cannot be evaluated fully using a single approach. To account for the varied mechanisms of antioxidant action, different types of antioxidant capacity measurements should be performed^32^. Hence, we employed different in-vitro assays to get a broader perspective on the antioxidant potential of this plant. APA (Sox) seed extract possesses a significant antioxidant potential as indicated by DPPH free radical scavenging with an IC_50_ value of 14.82 ± 0.85 µg/mL as compared to other extracts. Previously, reports have demonstrated that *A. precatorius* ethanol leaf extracts had antioxidant potential, with IC_50_ values of 33.37 μg/mL, 60.67 ± 1.03 µg/mL and 266.13 ± 1.2 µg/mL^32–34^. The antioxidant capacity of *A. precatorius* seed oil has also been demonstrated, with an IC_50_ value of 5.03 ± 0.24 mg/mL^35^. The radical scavenging activity can be explained by the different composition of each extract as there are compounds (polyphenolic flavonoids and phenolics) that react quickly with DPPH to get reduced due to the formation of nonradical^36^. As a virtue of their antioxidant properties, polyphenols and tannins may be helpful to human health, as evidenced by past research^37–39^.

Total antioxidant capacity was also measured using the FRAP assay, which revealed that the maximum FRAP value in APA (Mac) was 105,026.4 ± 57.50 mM Fe (II)/g DW and 95,601 ± 413.96 mM Fe (II)/g DW in APA (Sox), whereas previous studies found 8.91 ± 0.31 mg TEAC/g DW and 3.69 ± 0.13 mg AAE/g DW in ethanol seed extract, respectively^23^. This suggests that APA (Mac) has a strong ability to react with free radicals in order to change them into more stable non-reactive species and stop radical chain reactions.

We have reported for the first time the potential anticancer activity of *A. precatorius* seed extracts on Hep2C and HeLa cells as well as their interaction with cervical cancer receptors. All seed extracts exhibited antiproliferative activity in a dose-dependent manner. However, our results revealed that *A. precatorius* APE (Mac) was the most promising extract, with an IC_50_ value of 85.91 ± 6.7 µg/mL in the Hep2C cells, among others. Prior studies on Hep2C cells suggests that vulpinic acid, a major key phytocompound, has an IC_50_ value of 34.4 µM strongly suppresses cancer cell proliferation and acts as an anticancer agent through an underlying apoptotic mechanism^40^. Similarly, in HeLa cells, APA (Sox) with IC_50_ of 26.26 ± 1.09 µg/mL showed highly effective inhibitory activity as compared to the other extracts. Moreover, previous studies on HeLa Cells reported that ethyl acetate extract of *A. precatorius* roots had anticancer activity with an IC_50_ of 11.89 ± 0.63 µg/mL^41^. Since rutin and tannic acid were identified in extracts prepared by various methods, these were independently screened for antiproliferative activity using Hep2C and HeLa cells. In our study, we revealed that tannic acid had the maximum inhibitory activity against Hep2C and HeLa cell lines, whereas prior research had reported no data evaluating antiproliferative efficacies on Hep2C and HeLa cells, but had observed suppression of other cell lines^16^. Previous studies on rutin have shown the anticancer potential for a human renal cancer cell line (786-O) with an IC_50_ of 50 µM^42^.

This implies that polyphenolic flavonoids and tannins have anticancer properties through suppression of multiple oncogenic signalling pathways and tumor-promoting mechanisms^16^. However, to understand the exact mechanism of specificity against cervical cancer cells, further in-depth and extensive investigations are required.

Antioxidant enzymes such as GST, CAT and SOD catalyze the intracellular reduction of reactive oxygen species (ROS). Nevertheless, sometimes, antioxidant defence mechanisms are not sufficient to maintain a redox balance that promotes prooxidants. Oxidative stress occurs in the body, resulting in toxicity and genetic damage^43^.

The enzymatic activity and non-enzyme content were determined in Hep2C and HeLa cells. Our results revealed that in Hep2C cells, APE (Mac) extract revealed the highest SOD, catalase activity, GSH content, and the lowest MDA content, whereas APA (Mac) extract demonstrated the highest GST activity. Similarly, in HeLa cells, APA (Sox) extract showed the highest SOD, GST activity, GSH content, and the least MDA content, whereas APA (Mac) extract showed the highest catalase activity. Previous studies on extracts of *Ocimum sanctum* defatted seeds possessing polyphenolic flavonoids demonstrated its antioxidant enzyme activity in-vitro on Rat PC-12 cells^44^. However, no previous research on the antioxidant enzyme activity of *A. precatorius* seed extracts on cervical cancer cells has been done, to our knowledge. The effects of *A. precatorius* seed extracts on oxidative stress biomarkers could indicate that targeted interaction with the complex chain of cellular redox processes, such as increased antioxidant enzyme activity and decreased MDA levels in cervical cancer cells, can lead to an imbalance in antioxidant defence mechanisms, with a tendency toward pro-oxidants, a substrate for the cytotoxic effect.

To infer the role of the identified compounds and their association with cervical cancer receptors, we followed an in-silico pipeline. For this analysis, we studied crucial receptors like Glucocorticoids, HER2, VEGF and Hormonal Receptors (ER & PR) known to be associated with proliferation of cervical cancer^4–6^. Our in-silico investigations revealed that tannic acid showed the maximum binding energy against HER2 receptor and GCR compared to the standard (doxorubicin). Moreover, the literature cites the association of other important compounds like tangeretin, wogonin, quercetin, and other flavonoids that have shown lesser binding affinities with GCR and HER2^45,46^.

## Conclusion

The present study concludes that both the extraction methods (Maceration & Soxhlet) were effective in obtaining a maximum amount of biologically active compounds. We are among few studies that have looked at the anticancer activity of *A. precatorius* seed extracts on Hep2C and HeLa cells. This information is supported by HPLC quantification of rutin and tannic acid, which can be exploited to isolate bioactive components from *A. precatorius* seeds in the future. To the best of our knowledge, this is among the first reports to focus on the enzymatic and non-enzyme content in *A. precatorius* derived seed extracts.

Herein, we have also highlighted the association of a few identified compounds with crucial cervical cancer receptors, which had not been investigated earlier. Therefore, in line with the observations from this pilot study, our ongoing investigations are based on synthesizing of metal nanoparticles and checking their anticancer efficacy in cervical cancer cell lines. This approach is aimed at developing novel therapeutic strategies for cervical cancer management.

In summary, this plant possesses promising compounds to be tested as potential anticancer and antioxidant candidates for treatment of cervical cancer. However, further investigations need to be undertaken either to isolate the anticancer compounds or to determine the in-vivo biological activity of these extracts in order to promote them as potential cervical cancer models for preclinical trials.

## Methods

### Chemicals

The analytical grade chemicals were purchased from Hi-Media and Merck, India. Standard drugs were purchased from Sigma-Aldrich, India. 2,4,6-tris(2-pyridyl)-s-triazine TPTZ and MTT reagent were procured from Merck, India. Dulbecco's Modified Eagle's Medium (DMEM), fetal bovine serum (FBS) and penicillin (5000 U/mL), streptomycin (2500 U/mL) were purchased from Gibco (USA).

### Sample collection

Seeds of *A. precatorius* were collected from the Khari Baoli, Kucha Challan, Chandni Chowk, Delhi complies with relevant institutional, national, and international guidelines and legislation. Voucher specimens were deposited at Raw Material Herbarium and Museum, Delhi (RHMD), India and were authenticated by Dr. Sunita Garg, (Emeritus Scientist, CSIR-NISCAIR), with Ref No: NISCAIR/RHMD/Consult/2020/3697-98-2.

### Genomic DNA isolation and molecular identification

Genomic DNA was isolated from seeds of *A. precatorius* using Cetyltrimethylammonium bromide (CTAB) method^47^. The quality of DNA was observed through agarose gel electrophoresis.

The genomic DNA was amplified using *rbcLa*- forward primer (ATGTCACCACAAACAGAGACTAAAGC) and *rbcLa*- reverse primer (GTAAAATCAAGTCCACCRCG). PCR amplification was carried out in Veriti model of Applied Biosystem Thermo cycler^48^. The amplified product was then run on agarose gel and the appropriate band was sliced from the gel and was further processed for DNA elution using Gel Purification kit (Qiagen). The purified product was then utilized for sequencing using universal primers.

### Human cervical cell line culture

Human cervical cancer cell lines Hep2C and HeLa were obtained from National Centre for Cell Sciences (NCCS), Pune, India. The cells were cultured in Dulbecco’s modified Eagle’s medium (DMEM) supplemented with 10% (v/v) FBS, 100 U/ml penicillin, and 100 µg/mL streptomycin at 37 °C in a humidified atmosphere of 5% CO_2_.

### Preparation of extracts

Seeds were washed with distilled water to remove dirt and soil particles, followed by drying and grinding to form powder and used throughout the study.

### Soxhlet extraction method

Ethyl acetate and 70% ethanol were used to prepare *A.precatorius* seed extracts. 10 g of seed powder was placed inside a thimble made from thick filter paper and loaded into the soxhlet extractor. The soxhlet extractor was placed onto the flask containing the solvent (500 mL) equipped with a condenser. The extractor was then allowed to heat to reflux for 16 h at 70 °C. Extracts were filtered twice through a Whatman No.1 paper filter and concentrated to the dry mass with the aid of rotary evaporator^49^.

### Maceration extraction method

10 g of seed powder was soaked in 100 ml of solvent (Ethyl acetate and 70% Ethanol) and stored at room temperature for 7 days. The conical flasks of the extract were covered with cotton plugs to avoid the evaporation. After 7 days of incubation, they were filtered with muslin cloth followed by Whatman No.1 filter paper and concentrated to the dry mass with the aid of rotary evaporator^50^.

The dried extracts were dissolved in absolute dimethyl sulfoxide (DMSO) as 50 mg/mL and diluted with phosphate-buffered saline (PBS, pH 7.4) to give final concentrations.

### Quantification of phytochemical constituents

The total flavonoid content was measured by the Aluminium Chloride Spectrophotometric method. Absorbance was measured against the prepared blank at 510 nm and results were represented as quercetin equivalents (mg QE)/g of extract. Similarly, total phenolic content and total tannins content were quantified by the Folin–Ciocalteau method. Absorbance of the mixture was measured at 725 nm. Final results were represented as quercetin equivalents (mg QE)/g of extract and tannic acid equivalents (mg TA)/g of extract, respectively^44,49,51^. All the concentrations were calculated using a standard calibration plot.

The extracts were designated as APE (Mac); ethyl acetate extract obtained by maceration, APE (Sox); ethyl acetate extract obtained by soxhlet, APA (Mac); 70% ethanol extract obtained by maceration and APA (Sox); 70% ethanol extract obtained by soxhlet.

### Antioxidant activity

Antioxidant potential in the seed extracts was determined by electron transfer assay i.e. (2,2-Diphenyl-1-picrylhydrazyl) Radical scavenging assay (DPPH) and Ferric reducing antioxidant power (FRAP) assay. DPPH free radical scavenging assay was performed to measure the hydrogen donating or radical scavenging ability in a dose dependent manner at concentration (1–21 µg/mL) of quercetin and APA (Sox), (1–29 µg/mL) of APA (Mac), (150–170 µg/mL) of APE (Sox) and (150–650 µg/mL) of APE (Mac). Briefly, a 0.04 mM DPPH radical solution was prepared in methanol and then 900 μL of this solution was mixed with 100 μL of extract solution containing different concentrations of seed extracts. The absorbance was measured at 517 nm after 30 min of incubation. Methanol (95%) and DPPH solution were used as blank and control, respectively. Quercetin was used as the standard. 50% inhibitory concentrations (IC_50_ values) of the extracts were calculated from a graph as concentration versus percentage inhibition. Radical scavenging activity was expressed as the percentage of inhibition. Measurements were taken in triplicate. The IC_50_ of the extract and standards were determined graphically^52^.

The percentage of inhibition was calculated by using the formula:

$${\text{Percentage of inhibition}} = \left[ {\left( {{\text{absorbance of control}} - {\text{absorbance of reaction mixture}}} \right)/{\text{absorbance of control}}} \right] \times 100$$

Further, for the FRAP assay, FRAP reagent solution was prepared with 300 mM sodium acetate buffer (pH 3.6), 10 mM 2,4,6-tris(2-pyridyl)-*s*-triazine (TPTZ) in 40 mM HCl and 20 mM FeCl_3_·6H_2_O (10:1:1 v/v/v). The absorbance was measured at 593 nm after a 30 min incubation at room temperature against 50% ethanol as blank. A calibration curve was prepared using FeSO4 × 7H2O. FRAP values were expressed as mM Fe (II)/g dry weight of extract^49,53,54^.

### High pressure liquid chromatography

The presence of phenolic compounds and alkaloids in the prepared extracts was screened against standards (rutin, tannic acid and piperine) by HPLC. The analysis was performed using a C-18 reversed phase column (Phenomenex, Gemini 5 μ, 250 mm length × 4.6 mm internal diameter). The mobile phase consisted of methanol: 0.1% orthophosphoric acid (77:23) for rutin, methanol: water (50:50) for tannic acid and 1% acetic Acid: acetonitrile (52:48) for piperine were chosen for the separation at a constant flow rate of 1 mL/min. The column temperature was set to 38 °C and the injection volume was 2 μL. The wavelengths were set to 370 nm for the detection of rutin, 280 nm for tannic acid, and 343 nm for piperine. To plot the standard calibration curves, standard stock solutions of rutin, tannic acid, and piperine were produced in methanol at various concentrations (5–100 µg/mL). The results were expressed as milligrams of each compound per g of dry weight (DW) of the extract^49^.

### Antiproliferative activity

Cytotoxic activity of *A. precatorius* seed extracts were evaluated using a modified 3-(4, 5-dimethylthiazol-2-yl)-2, 5-diphenyltetrazolium (MTT) assay on Hep2C and HeLa cells^55^. Briefly, cells were seeded (~ 1 × 10^4^ cells/well) into flat-bottomed 96-well culture plates and incubated for 24 h at 37 °C using 5% CO_2_ and 95% air. Hep2C cells were treated with doxorubicin (0.1–10 μg/mL), tannic acid (5–45 μg/mL), rutin (5–50 μg/mL), APE (Mac/Sox) (50–200 μg/mL) and APA (Mac/Sox) (50–450 μg/mL)^56^. Further, HeLa cells were treated with doxorubicin (0.39–1.56 μg/mL), tannic acid (5–20 μg/mL), rutin (5–40 μg/mL), APE (Mac/Sox) (5–150 μg/mL) and APA (Mac) (5–100 μg/mL)/APA (Sox) (5–50 μg/mL). After 48 h of incubation, 10 μl of MTT reagent (5 mg/mL) was added and mixtures were reincubated for 3 h. The resulting formazan was solubilized with DMSO (100 μl). Finally, the absorbance of formazan was measured at 570 nm using an automated microplate reader (Bio-Rad, Illinois, USA). Experiments were carried out in triplicates. The cell viability (%) was obtained by comparing the absorbance between the samples and the negative control^57^.

The percent inhibition was calculated by using the following formula:

$$\% \,{\text{Inhibition}} = {1}00{-}\left( {{\text{mean OD of test compound}} - {\text{mean OD of negative control}}} \right)/\left( {{\text{mean OD of positive control}} - {\text{mean OD of negative control}}} \right) \times {1}00$$

### Antioxidant enzyme activity assay on Hep2C and HeLa cells

To estimate the effect of *A. precatorius* seed extracts on Hep2C and HeLa cells, the enzymatic activity [superoxide dismutase (SOD), catalase (CAT), Glutathione-S-Transferase (GST)] and non-enzyme content [Glutathione content (GSH) and lipid peroxidation (Malondialdehyde (MDA) content)] were evaluated. A total of 1.51 × 10^5^ cells/well were seeded in 24 well plates and incubated for 24 h in a CO_2_ incubator at 37 °C followed by a 48 h treatment with the obtained IC_50_ specific values of each of these extracts. The cells were harvested by washing with PBS followed by trypsinization. Further, the samples were centrifuged at 10,000 rpm for 20 min at 4 °C to obtain the cell lysate which was further used for estimation of enzyme and non-enzyme content in accordance with standardized procedures^44^.

### Molecular docking

#### AutoDock/vina docking

Three-dimension SDF files of rutin (polyphenolic flavonoids), tannic acid and doxorubicin were downloaded from Pubchem database (https://pubchem.ncbi.nlm.nih.gov/). PDB files of Estrogen (PDB ID: 1ERR), Glucocorticoid (PDB ID: 1M2Z), HER 2 (PDB ID: 3PP0), Progesterone (PDB ID: 1SQN) and VEGF (PDB ID: 1FLT) were downloaded from Protein data bank (https://www.rcsb.org). PDBQT files and grid box creations were prepared using Graphical User Interface program AutoDock Tools (ADT). ADT introduced polar hydrogen, united atom Kollman charges to the proteins and the files were saved in PDBQT format. AutoGrid was used for the preparation of the grid map using a grid box. AutoDock/Vina was employed for docking using protein and ligand information along with grid box properties in the configuration file^58^. The outcomes under 1.0 Å in positional root-mean-square deviation (RMSD) were clustered together and addressed by the free energy of binding. The pose with the least energy of binding or binding affinity was removed and aligned with receptor structure for additional examination. Interaction of amino acids with molecules were observed by Discovery studio 2021 and a 2-D diagrams of the interactions were saved.

### Statistical analysis

The statistical data were represented as mean ± standard deviation (SD) from three independent experiments. Statistical analysis was performed using one-way analysis of variance (ANOVA) followed by Tukey's multiple comparisons test. A probability value of ≤ 0.05 was considered as statistically significant. All analyses were performed using GraphPad Prism version 8.0.2.

## Supplementary Information

Supplementary Information.

## Data Availability

All data are available in the manuscript.
