# Supplementary material for: In-vitro antiproliferative efficacy of Abrus precatorius seed extracts on cervical carcinoma
Source: Sci Rep. 2022 Jun 17;12:10226. doi: 10.1038/s41598-022-13976-7 (PMC9205867; doi:10.1038/s41598-022-13976-7)
Supplement: Supplementary file 1 — Supplementary Information. [file 41598_2022_13976_MOESM1_ESM.docx]

Graph 1: Standard curve of quercetin (20-100µg/ml) for total flavonoid content.

Graph 2: Standard of quercetin (20-100 µg/ml) for total phenolic content.

Graph 3: Standard of tannic acid (20-100 µg/ml) for total tannin content.


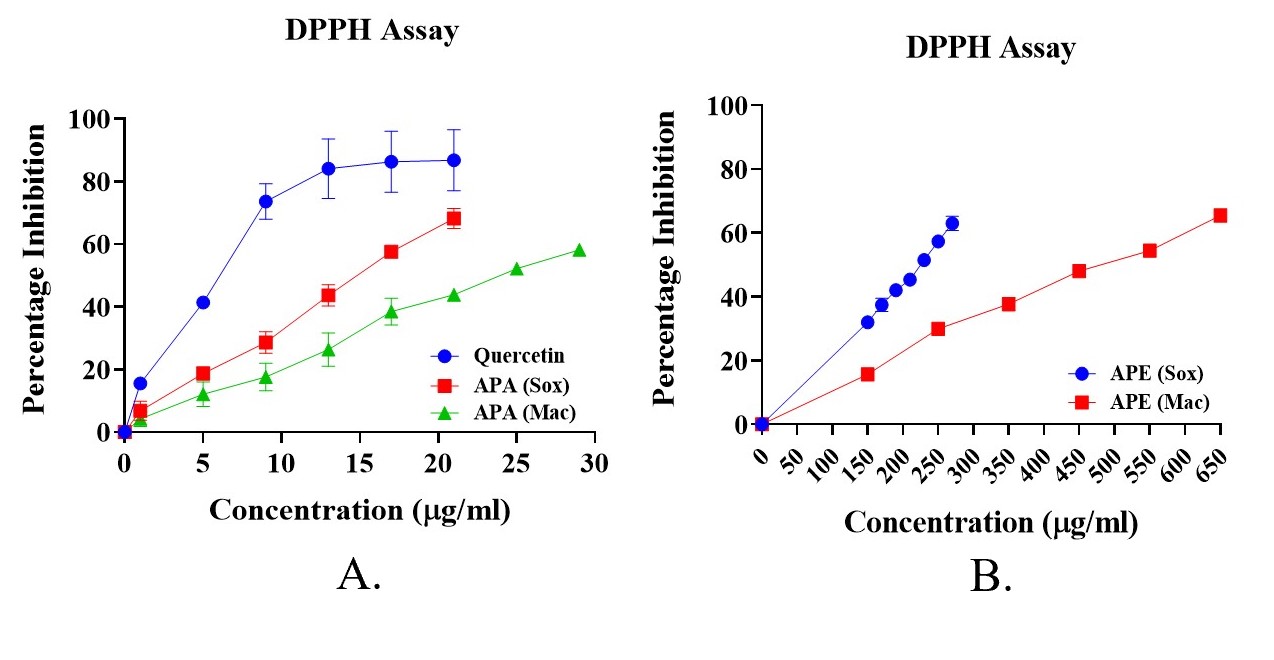
Graph 4. Determination of a DPPH radical scavenging activity of *A. precatorius* seed extracts at concentration; **(A)** (1 – 21 µg/mL) of quercetin / APA (Sox) and (1 - 29 µg/mL) of APA (Mac); **(B)** (150 – 170 µg/mL) of APE (Sox) and (150 – 650 µg/mL) of APE (Mac). All experiments were performed in triplicate. Data are expressed as mean ± SD (n = 3), for all tested dosages

Graph 5: Standard calibration curve of FeSO_4_ for FRAP assay.

Graph 6: Standard curve of rutin (5-100 µg/ml) by HPLC.

Graph 7: Standard curve of tannic acid (5-100 µg/ml) by HPLC.

Graph 8: Standard curve of piperine (5-100 µg/ml) by HPLC.

A. B.

Graph 9: Growth inhibition curves on Hep2C cell lines are determined by MTT assay for 48 h of treatment with; (**A)** Doxorubicin (0.1 - 10 μg/mL), tannic acid (5 - 45 μg/mL) and rutin (5 - 50 μg/mL); (**B**) APE (Mac/ Sox) (50 - 200 μg/mL) and APA (Mac/ Sox) (50 - 450μg/mL). Data are shown as mean ± SD of three independent experiments.

A. B.

Graph 10: Growth inhibition curves on HeLa cell lines are determined by MTT assay for 48 h of treatment with; (**A)** Doxorubicin (0.39 - 1.56 μg/mL), tannic acid (5 - 20 μg/mL), rutin (5 - 40 μg/mL); (**B**) APE (Mac/ Sox) (5 - 150 μg/mL) and APA (Mac) (5 - 100 μg/mL) / APA (Sox) (5 - 50μg/mL) on the HeLa cells growth after 48 h.
